# Supplementary material for: Phylogeographic reconstruction of the marbled crayfish origin
Source: Commun Biol. 2021 Sep 17;4:1096. doi: 10.1038/s42003-021-02609-w (PMC8448756; doi:10.1038/s42003-021-02609-w)
Supplement: Supplementary file 2 — Supplementary Material [file 42003_2021_2609_MOESM2_ESM.pdf]

Supplementary Material

Phylogeographic reconstruction of the marbled crayfish origin

Contents:

Supplementary Figures S1-S5

Supplementary Tables S1-S3

Supplementary References

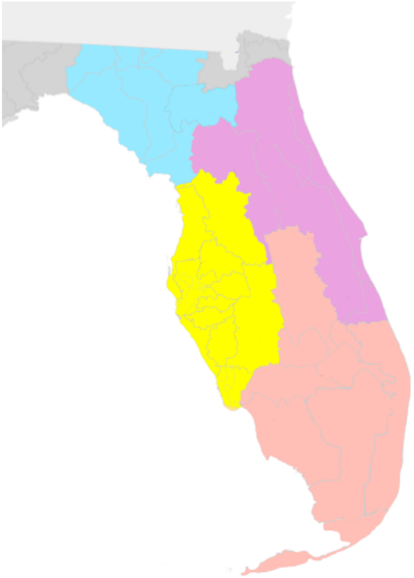

Fig. S1. The four major water catchment areas of Florida: Suwannee River (blue), St. John's River (purple), Southwest (yellow) and Everglades (red). The areas represent the subregions defined by 4-digit hydrologic unit codes[1]. The headwater wetlands of the upper St. John's River (southern part of the purple region) and the Everglades were historically well connected.

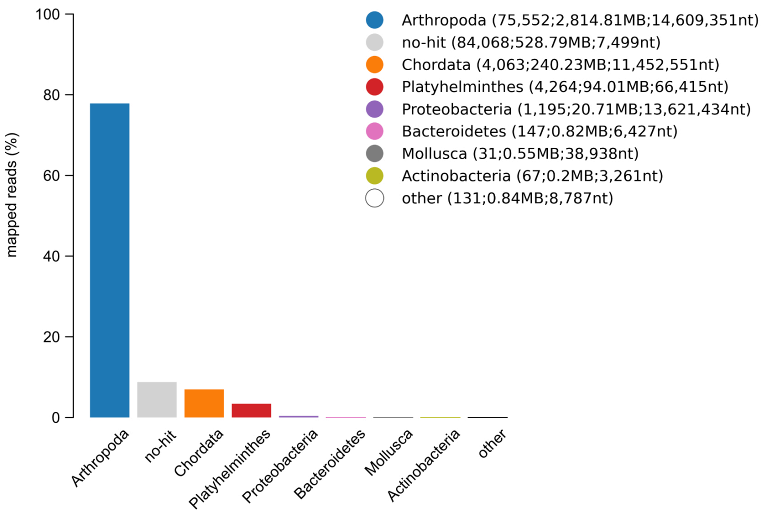

Fig. S2. Taxonomic interrogation of the *Procambarus virginalis* genome assembly. Mapping of a representative *P. virginalis* WGS dataset resulted in 97.4% mapped reads. Bars represent the taxonomic classification, based on the entire BLAST nucleotide database. The legend indicates the number, total span and N50 of sequences.

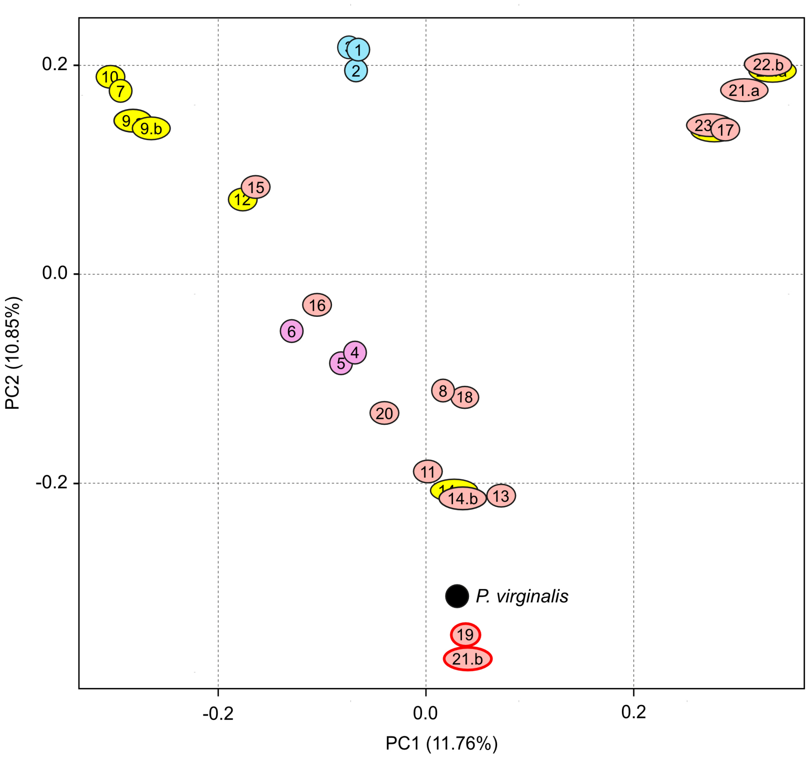

Fig. S3. Principal component analysis, based on a combined *Procambarus fallax* and *Procambarus virginalis* WGS dataset. Colors indicate the four major subpopulations: Suwannee (blue), St. John's (purple), Southwest (yellow) and Everglades (red). Animals with a particularly closely related to *P. virginalis* mitochondrial genome sequence are highlighted by red circles. Compared to Fig. 2, this PCA shows considerably less clusterization, which is due to the substantial reduction in the number of linkage-pruned variant sites (N=15,774). The number of common variant sites is limited by the clonality of the *P. virginalis* genomes and the resulting low number of SNVs between the *P. virginalis* sample (MA2, ref. 2) and the reference genome.

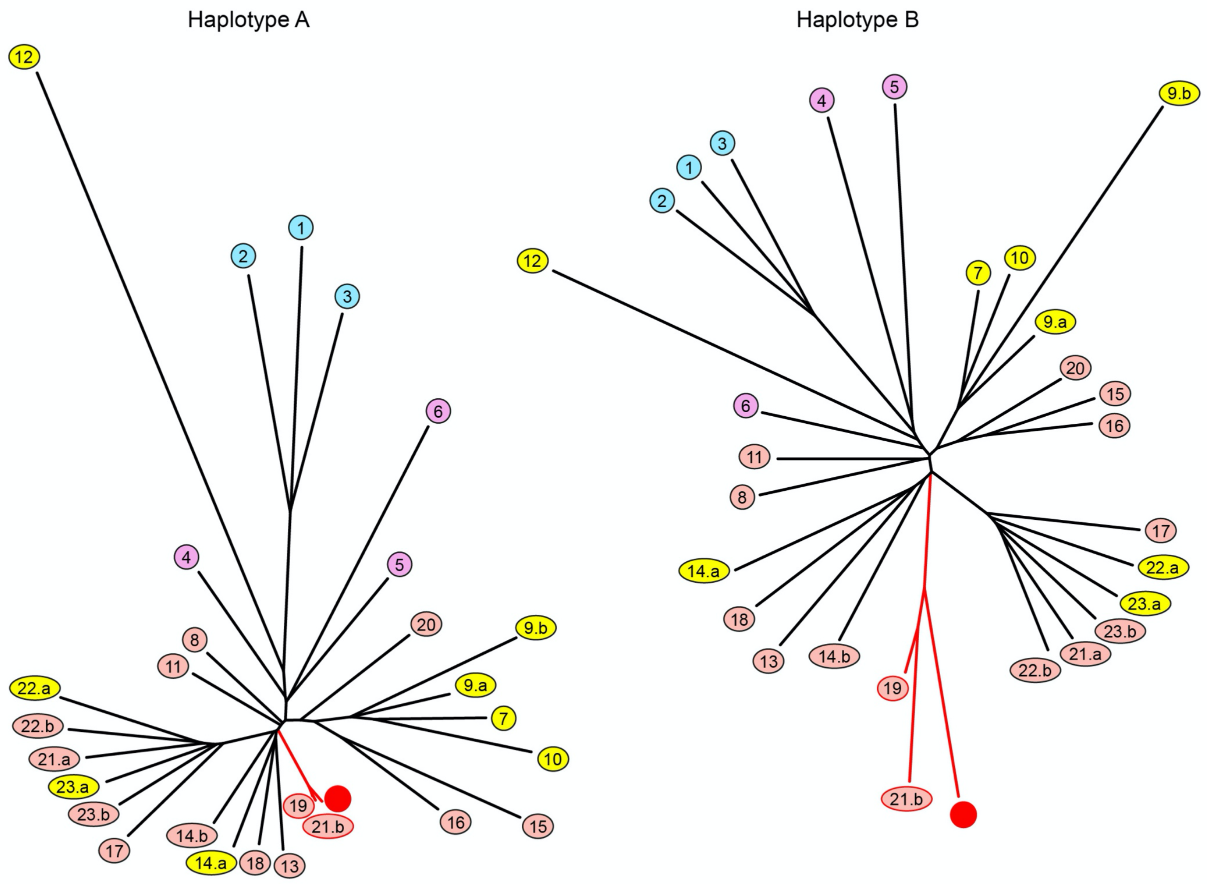

Fig. S4. Nuclear haplotype relationship analysis between *Procambarus fallax* and *P. virginalis*. Neighbour-joining tree for the *P. virginalis* majority ("A") haplotype and minority ("B") haplotype. *P. virginalis* is indicated by a bright red dot, scale bars indicate counts. Colors indicate the four major subpopulations: Suwannee (blue), St. John's (purple), Southwest (yellow) and Everglades (red).

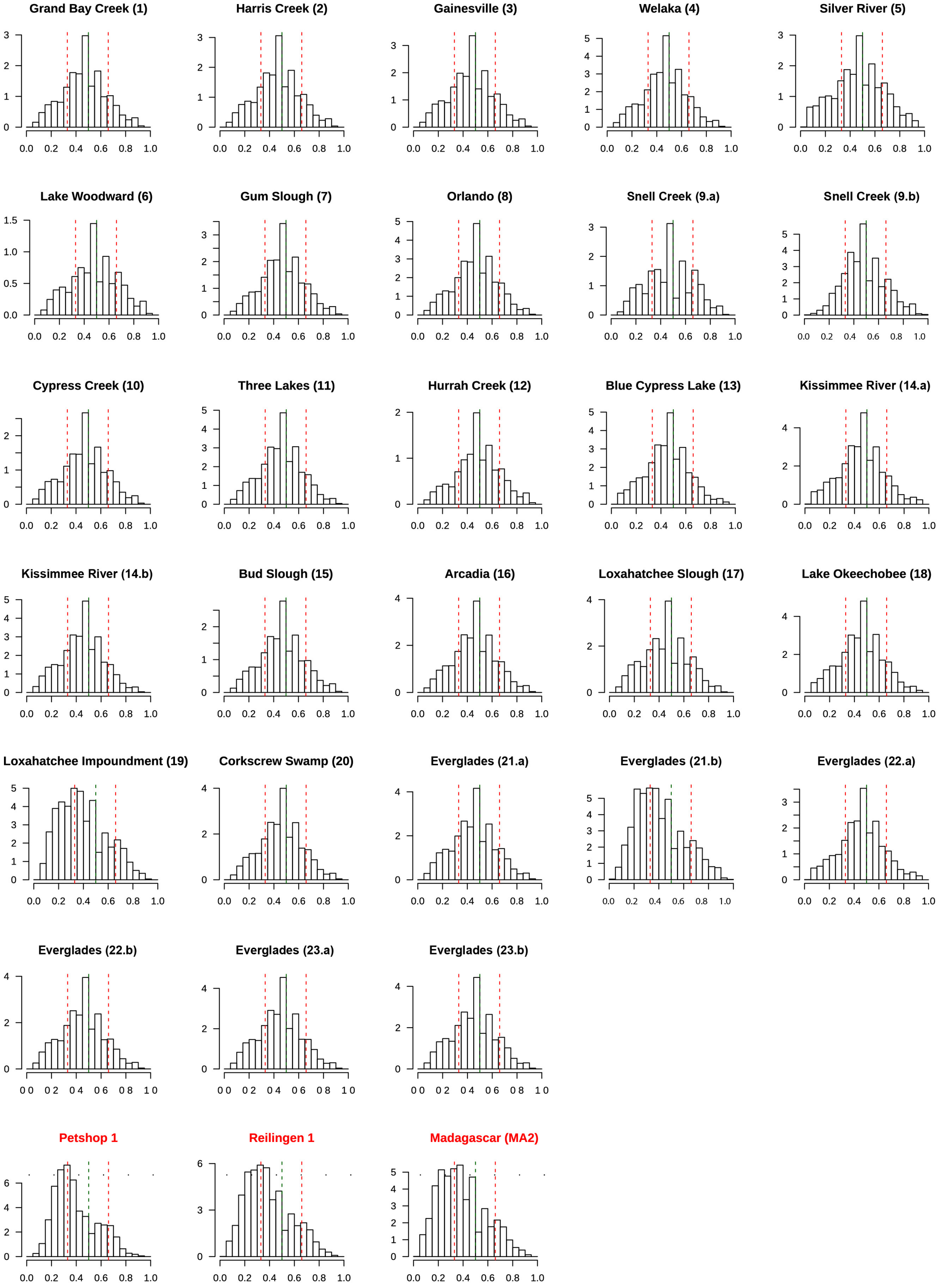

Fig. S5. Heterozygous allele frequency distribution for the complete (N=28) *Procambarus fallax* whole-genome sequencing dataset. The histograms show the frequencies of heterozygous alternative allele ratios. Numbers for allele frequencies are indicated in 10^5. Dashed vertical lines correspond to allele frequencies commonly observed in triploid (0.33 and 0.66) and diploid (0.5) organisms. The results from three independent *P. virginalis* (highlighted in red) genomes[2] are included for comparison.

Table S1. Overview of *P. fallax* collection sites.

#	Name	Location	Collection
1	Grand Bay Creek	30°54'09.4"N 83°05'36.8"W	C.E.S., June 2018
2	Harris Creek	30°38'24.2"N 82°03'23.2"W	C.E.S., April 2018
3	Gainesville	29°47'49.6"N 82°20'07.5"W	C.E.S., June 2018
4	Welaka	29°28'06.7"N 81°45'11.1"W	C.E.S., June 2018
5	Silver River	29°12'07.2"N 82°00'35.3"W	ref. 3
6	Lake Woodward	28°49'13.0"N 81°40'54.3"W	N.J.D., March 2019
7	Gum Slough	28°41'49.6"N 82°14'39.8"W	C.E.S., June 2018
8	Orlando	28°25'32.5"N 81°09'40.6"W	C.E.S., March 2019
9	Snell Creek	28°07'57.2"N 81°32'30.2"W	C.E.S., March 2019
10	Cypress Creek	28°05'17.2"N 82°24'27.1"W	C.E.S., March 2019
11	Three Lakes	27°55'48.5"N 81°07'36.1"W	N.J.D., March 2019
12	Hurrah Creek	27°45'45.4"N 82°08'47.0"W	C.E.S., March 2019
13	Blue Cypress Lake	27°41'47.1"N 80°42'43.1"W	ref. 4
14	Kissimmee River	27°29'07.4"N 81°11'57.5"W	N.J.D., January 2018
15	Bud Slough	27°16'03.7"N 82°07'05.5"W	C.E.S., March 2019
16	Arcadia	27°12'31.5"N 81°49'56.5"W	C.E.S., March 2019
17	Loxahatchee Slough	26°50'48.5"N 80°10'13.2"W	N.J.D., April 2018
18	Lake Okeechobee	26°50'33.4"N 80°59'52.2"W	N.J.D., February 2018
19	Loxahatchee Impoundments	26°29'24.5"N 80°13'14.9"W	N.J.D., April 2018*
20	Corkscrew Swamp	26°22'29.1"N 81°36'32.8"W	N.J.D., April 2018
21	Everglades	26°04'12.7"N 80°40'37.6"W	N.J.D., October 2017
22	Everglades	25°46'04.2"N 80°40'23.5"W	N.J.D., November 2017
23	Everglades	25°45'43.0"N 80°30'08.2"W	N.J.D., November 2017

*second collection (19.1-3) in September 2019. C.E.S.: Christopher E. Skelton; N.J.D.: Nathan J. Dorn.

Table S2. *Procambarus fallax* whole-genome sequencing overview.

#	name	sex	sequencing protocol	raw read pairs	processed read pairs	mapped (%)	covg. (X)
1	Grand Bay Creek	f	HiSeqX PE150	423,783,854	337,745,407	82.9	27.2
2	Harris Creek	m	HiSeqX PE150	400,883,515	309,059,581	81.8	24.9
3	Gainesville	f	HiSeqX PE150	382,980,427	304,421,473	80.3	23.9
4	Welaka	m	HiSeqX PE150	419,925,777	338,428,570	82.3	26.7
5	Silver River	m	HiSeqX PE150	515,703,320	325,029,936	78.8	25.7
6	Lake Woodward	f	HiSeqX PE150	401,671,902	296,294,299	66.9	19.8
7	Gum Slough	m	HiSeqX PE150	429,448,611	339,413,271	80.4	26.7
8	Orlando	f	HiSeqX PE150	404,630,133	313,607,100	69.7	22.2
9.a	Snell Creek	m	HiSeqX PE150	405,889,320	182,146,721	64.2	12.3
9.b	Snell Creek	f	HiSeqX PE150	439,200,494	331,877,749	79.0	23.5
10	Cypress Creek	f	HiSeqX PE150	403,530,773	303,556,280	77.8	23.5
11	Three Lakes	f	HiSeqX PE150	401,945,296	310,359,901	81.0	24.4
12	Hurrah Creek	m	HiSeqX PE150	399,391,466	301,198,204	82.0	24.4
13	Blue Cypress Lake	m	HiSeqX PE150	428,390,609	332,127,247	82.5	25.4
14.a	Kissimmee River	f	HiSeqX PE150	408,765,829	324,407,550	83.4	25.9
14.b	Kissimmee River	f	HiSeqX PE150	438,654,151	356,033,853	78.2	27.0
15	Bud Slough	m	HiSeqX PE150	398,886,536	302,415,181	82.5	24.6
16	Arcadia	m	HiSeqX PE150	403,267,452	313,879,146	78.4	24.4
17	Loxahatchee Slough	f	HiSeqX PE150	419,442,289	208,892,712	85.2	18.1
18	Lake Okeechobee	m	HiSeqX PE150	388,988,492	299,392,496	81.4	23.4
19	Loxahatchee Imp.	f	HiSeqX PE150	390,255,144	308,958,034	86.5	25.8
20	Corkscrew Swamp	f	HiSeqX PE150	383,000,736	302,318,143	84.4	25.4
21.a	Everglades	f	HiSeqX PE150	427,850,688	336,421,204	82.8	26.8
21.b	Everglades	f	HiSeqX PE150	513,457,416	370,729,883	84.6	30.4
22.a	Everglades	n.d.	HiSeqX PE150	433,735,326	347,901,252	83.1	28.0
22.b	Everglades	n.d.	HiSeqX PE150	424,902,716	339,802,442	82.9	27.2
23.a	Everglades	n.d.	HiSeqX PE150	432,856,065	343,689,994	81.8	24.9
23.b	Everglades	n.d.	HiSeqX PE150	423,783,854	294,515,468	80.3	23.9

Sequencing coverages (covg.) were calculated for the *P. virginalis* V1.0 genome reference sequence. n.d., not determined.

Table S3. Key features of the new *Procambarus virginalis* genome assembly version.

**Feature**	**V1.0 (this study)**	**V0.4 (ref. 4)**
total scaffolds	169,515	3,394,710
total assembly length	3,700,955,717 bp	3,511,656,756 bp
max sequence length	73,605,948 bp	717,999 bp
mean seq. length	21,832 bp	1,034 bp
N50	144,428 bp	29,652 bp
GC content	44.32%	43.31%
Number of gap bases (N)	663,440,479 bp	1,662,662,583 bp

Supplementary References

1. Mylavarapu R., Hines K., Obreza T., Means G. Watersheds of Florida: Understanding a Watershed Approach to Water Management. *EDIS*, SL367 (2017).

2. Gutekunst J., et al. Clonal genome evolution and rapid invasive spread of the marbled crayfish. *Nat. Ecol. Evol.* **2**, 567-573 (2018).

3. Manteuffel-Ross T. M., Stolen E., Hinkle C. R. Abundance and habitat associations of two Florida crayfishes, Procambarus paeninsulanus (Faxon, 1914) and P. fallax (Hagen, 1870) (Decapoda: Astacoidea), assessed with N-mixture modeling. *Journal of Crustacean Biology* **38**, 285–294 (2018).

4. Levy T., Rosen O., Simons O., Savaya Alkalay A., Sagi A. The gene encoding the insulin-like androgenic gland hormone in an all-female parthenogenetic crayfish. *PLoS ONE* **12**, e0189982 (2017).
