# Supplementary material for: Phylogeographic reconstruction of the marbled crayfish origin
Source: Commun Biol. 2021 Sep 17;4:1096. doi: 10.1038/s42003-021-02609-w (PMC8448756; doi:10.1038/s42003-021-02609-w)
Supplement: Supplementary file 3 — Reporting Summary [file 42003_2021_2609_MOESM3_ESM.pdf]

nature research

Corresponding author(s): Frank Lyko

Last updated by author(s): Jul 19, 2021

Reporting Summary

Nature Research wishes to improve the reproducibility of the work that we publish. This form provides structure for consistency and transparency in reporting. For further information on Nature Research policies, see our Editorial Policies and the Editorial Policy Checklist.

Statistics

For all statistical analyses, confirm that the following items are present in the figure legend, table legend, main text, or Methods section.

n/a	Confirmed	
☐	☒	The exact sample size (*n*) for each experimental group/condition, given as a discrete number and unit of measurement
☐	☒	A statement on whether measurements were taken from distinct samples or whether the same sample was measured repeatedly
☐	☒	The statistical test(s) used AND whether they are one- or two-sided<br>*Only common tests should be described solely by name; describe more complex techniques in the Methods section.*
☒	☐	A description of all covariates tested
☐	☒	A description of any assumptions or corrections, such as tests of normality and adjustment for multiple comparisons
☐	☒	A full description of the statistical parameters including central tendency (e.g. means) or other basic estimates (e.g. regression coefficient) AND variation (e.g. standard deviation) or associated estimates of uncertainty (e.g. confidence intervals)
☐	☒	For null hypothesis testing, the test statistic (e.g. *F*, *t*, *r*) with confidence intervals, effect sizes, degrees of freedom and *P* value noted<br>*Give P values as exact values whenever suitable.*
☒	☐	For Bayesian analysis, information on the choice of priors and Markov chain Monte Carlo settings
☒	☐	For hierarchical and complex designs, identification of the appropriate level for tests and full reporting of outcomes
☒	☐	Estimates of effect sizes (e.g. Cohen's *d*, Pearson's *r*), indicating how they were calculated

Our web collection on statistics for biologists contains articles on many of the points above.

Software and code

Policy information about availability of computer code

Data collection

For data collection documented Illumina and PacBio sequencing protocols and data collection algorithms were used as stated in the manuscript

Data analysis

All software packages (including details, such as version numbers) are provided and routines are explained in the Methods section of the manuscript or in the Supplementary Information. Following software and packages and version numbers were used for data analysis:
Canu, v1.7
L_RNA_SCAFFOLDER, v1.0
MAKER, v3.00
InterproScan, v5.39-77.0
BUSCO, v4.1.4
blobtools, v1.1.1
PLINK, v1.9
RepeatModeler, v2.0.1
RepeatMasker, v4.1.1
Trimmomatic, v0.32
bowtie2, v2.1.0
samtools, v1.9
freebayes, v0.9.21-7-g7dd41db
R, v3.6.1
R Bioconductor package ggplot2, v3.2.1
R Bioconductor package ape, v5.3

For manuscripts utilizing custom algorithms or software that are central to the research but not yet described in published literature, software must be made available to editors and reviewers. We strongly encourage code deposition in a community repository (e.g. GitHub). See the Nature Research guidelines for submitting code & software for further information.

Data

Policy information about availability of data

All manuscripts must include a data availability statement. This statement should provide the following information, where applicable:

- Accession codes, unique identifiers, or web links for publicly available datasets
- A list of figures that have associated raw data
- A description of any restrictions on data availability

All sequencing data have been deposited as an NCBI BioProject (accession number PRJNA587442).

Field-specific reporting

Please select the one below that is the best fit for your research. If you are not sure, read the appropriate sections before making your selection.

☐ Life sciences ☐ Behavioural & social sciences ☒ Ecological, evolutionary & environmental sciences

For a reference copy of the document with all sections, see nature.com/documents/nr-reporting-summary-flat.pdf

Ecological, evolutionary & environmental sciences study design

All studies must disclose on these points even when the disclosure is negative.

Study description	Our study provides a population-scale phylogeographic analysis of Procambarus fallax to reconstruct the origin of the marbled crayfish (Procambarus virginalis). Our results show that both parental haplotypes of the triploid P. virginalis were inherited from the Everglades subpopulation of P. fallax. Comprehensive whole-genome sequencing also detected triploid P. fallax specimens in the same population.
Research sample	Procambarus fallax were collected within the known native range of the species (Florida and Southern Georgia).
Sampling strategy	Samples were collected with handnets. Multiple samplings were performed at individual sites and animals were taxonomically classified, sexed and then randomly chosen for further analysis.
Data collection	Data was collected during or immediatedly after sampling as handwritten notes.
Timing and spatial scale	Animals were collected between October 2017 and March 2019.
Data exclusions	No data were excluded.
Reproducibility	Whole-genome sequencing was performed once per sample.
Randomization	Samples from populations were taken without specific selection.
Blinding	Persons performing sample preparation and WGS were unaware of the sample identity.

Did the study involve field work? ☒ Yes ☐ No

Field work, collection and transport

Field conditions	As this is a genomic study, field conditions are not relevant.
Location	Provided in the Supplementary Table S2.
Access & import/export	Public waterbodies, accessed by walking. Collections were performed in compliance with state and local regulations.
Disturbance	There were no disturbances.

Reporting for specific materials, systems and methods

We require information from authors about some types of materials, experimental systems and methods used in many studies. Here, indicate whether each material, system or method listed is relevant to your study. If you are not sure if a list item applies to your research, read the appropriate section before selecting a response.

Materials & experimental systems

n/a	Involved in the study
☒	☐ Antibodies
☒	☐ Eukaryotic cell lines
☒	☐ Palaeontology and archaeology
☐	☒ Animals and other organisms
☒	☐ Human research participants
☒	☐ Clinical data
☒	☐ Dual use research of concern

Methods

n/a	Involved in the study
☒	☐ ChIP-seq
☒	☐ Flow cytometry
☒	☐ MRI-based neuroimaging

Animals and other organisms

Policy information about studies involving animals; ARRIVE guidelines recommended for reporting animal research

Laboratory animals	Not applicable.
Wild animals	Procambarus fallax, mixed sex, approx. 2 years old. Animals were caught by handnets and killed by decapitation after catching.
Field-collected samples	Not applicable.
Ethics oversight	Not applicable (invertebrate species).

Note that full information on the approval of the study protocol must also be provided in the manuscript.
